# Computational study on novel natural inhibitors targeting BCL2

**DOI:** 10.1007/s12032-021-01513-x

**Published:** 2021-07-14

**Authors:** Xiaye Lv, Yuting Jiang, Xinhui Wang, HaoQun Xie, Gaojing Dou, Jing Wang, Wenzhuo Yang, Hongyu Wang, Zijian Li, Xiangheng Zhang, Zhenghe Chen

**Affiliations:** 1 Department of Neurosurgery, Sun Yat-Sen University Cancer Center, Guangzhou, 510060 China; 2 State Key Laboratory of Oncology in South China, Sun Yat-Sen University Cancer Center, Guangzhou, People’s Republic of China; 3 Collaborative Innovation Center for Cancer Medicine, Guangzhou, China; 4 Department of Hematology, The First Clinical Medical School of Lanzhou University, No.1, Donggangxi Rd, Chengguan District, Lanzhou, 730000 Gansu China; 5 Clinical College, Jilin University, Street Xinmin 70, Changchun, China; 6 Department of Oncology, First People’s Hospital of Xinxiang, Street Yiheng 63, Xinxiang, China; 7 Department of Breast Surgery, The First Hospital of Jilin University, Street Xinmin 71, Changchun, China

**Keywords:** BCL2, Drug treatment, Inhibitor, Virtual screening

## Abstract

Ideal lead compounds and candidate drugs with inhibitory effect on BCL2 were screened from ZINC database, which laid a foundation for drug development and compound improvement of drug treatment for diffuse large B-cell lymphoma (DLCBL). Identification of potential BCL2 inhibitors by computer-aided virtual screening. Libdock was applied to 17,931 compounds and the top 20 were selected for further analysis. Selected compounds were performed absorption, distribution, metabolism, and excretion (ADME) and toxicity prediction. The binding affinity between the selected ligands and BCL2 was confirmed by Molecular docking. The new natural compounds, ZINC00000255131 and ZINC00013298233, were found to bind closely with BCL2. Furthermore, they all scored lower in ames-induced mutagenicity, rodent carcinogenicity, non-developmental toxicity potential, and cytochrome P4502D6 tolerance. Molecular dynamics simulation shows that the combinations of ZINC00000255131 and ZINC00013298233 with BCL2 in the natural environment are more stable. Two new compounds, ZINC00000255131 and ZINC00013298233, were found to be potential inhibitors of BCL2. These compounds have been proved to be safe, which is of great significance for the development and improvement of DLCBL drugs.

## Introduction

Diffuse large B-cell lymphomas (DLCBL) is one of the most common malignant lymphomas in adults, which account for 24% of new cases in non-Hodgkin lymphoma [1]. This disease is highly aggressive and needs treatment rapidly. The most common early treatment is chemotherapy with R-CHOP (rituximab, cyclophosphamide, doxorubicin, vincristine, and prednisone), which can cure about 50–60% patients [2, 3]. But unfortunately, patients that are refractory to early treatment or relapse after remission have particularly poor prognosis [4].

Research shows that DLCBL is highly related to BCL2, an oncogene on chromosome 18q21 with antiapoptotic properties and has potent antiapoptotic functions [5–7]. Oxidative stress, genomic instability, and other damage induce the expression of BH3 family proteins in normal lymphocytes, whose function is to inhibit BCL2 and enable cells to enter the apoptotic program [8, 9]. The mechanism of BCL-2 family proteins triggering apoptosis is through the formation of pores in the outer membrane of mitochondria [10]. BCL-2 protein mainly controls cell apoptosis by regulating the direct binding interaction of mitochondrial outer membrane permeabilization (MOMP), which resulting in irreversible release of intermembrane space proteins, and followed by caspase activation and apoptosis [11, 12]. MOMP causes the release of pro-apoptotic factors such as cytochrome from the mitochondrial membrane space (IMS) into the cytosol. Subsequently, the caspase cascade is activated and eventually the cells are destroyed [11, 13, 14].

Bcl-2 protein can inhibit apoptosis, which promotes the survival of cancer cells and the generation of chemoresistance [15, 16]. The BCL2 inhibitor approved by the regulatory agency is Obatoclax, whose indications are narrow and mainly targeted at patients with recurrent chronic lymphocytic lymphoma/leukemia [17–19]. In most common B-cell malignancies, existing BCL2 inhibitors show only moderate clinical activity [20]. Therefore, we need more effective BCL2 targeted drugs.

In this study, a series of structural biology and chemical methods have been used to screen and identify clues leading to compounds with potential inhibitory functions associated with BCL2. In addition, our research also predicted the absorption, distribution, metabolism, excretion and toxicity of certain candidate compounds. A list of candidate drugs and their pharmacological properties were obtained from the ZINC15 database. These lists can provide a solid basis for BCL2 inhibitor development research. The significance of this research is to find lead compounds for BCL2 inhibitors, which lay the foundation for drug development and compound improvement in cancer drug treatment.

## Methods and materials

### Discovery Studio software and ligand library

Discovery Studio 4.5 software (BIOVIA, San Diego, California, USA) is a suite of software for simulating small molecule and macromolecule systems. It aims to provide protein modeling, optimization, and drug design tools by applying protein structure and structural biologic computation. LibDock module of Discovery Studio was employed for virtual screening. The CDOCKER module was used for docking study. The ADME module was analyzed for pharmacologic properties. The Natural Products database in the ZINC15 database was selected to screen STING agonists. The ZINC15 database is a free database of commercially available compounds provided by the Irwin and Shoichet Laboratories, Department of Pharmaceutical Chemistry, University of California, San Francisco (San Francisco, California, USA).

### Structure-based virtual screening using LibDock

**Fig. 1:**
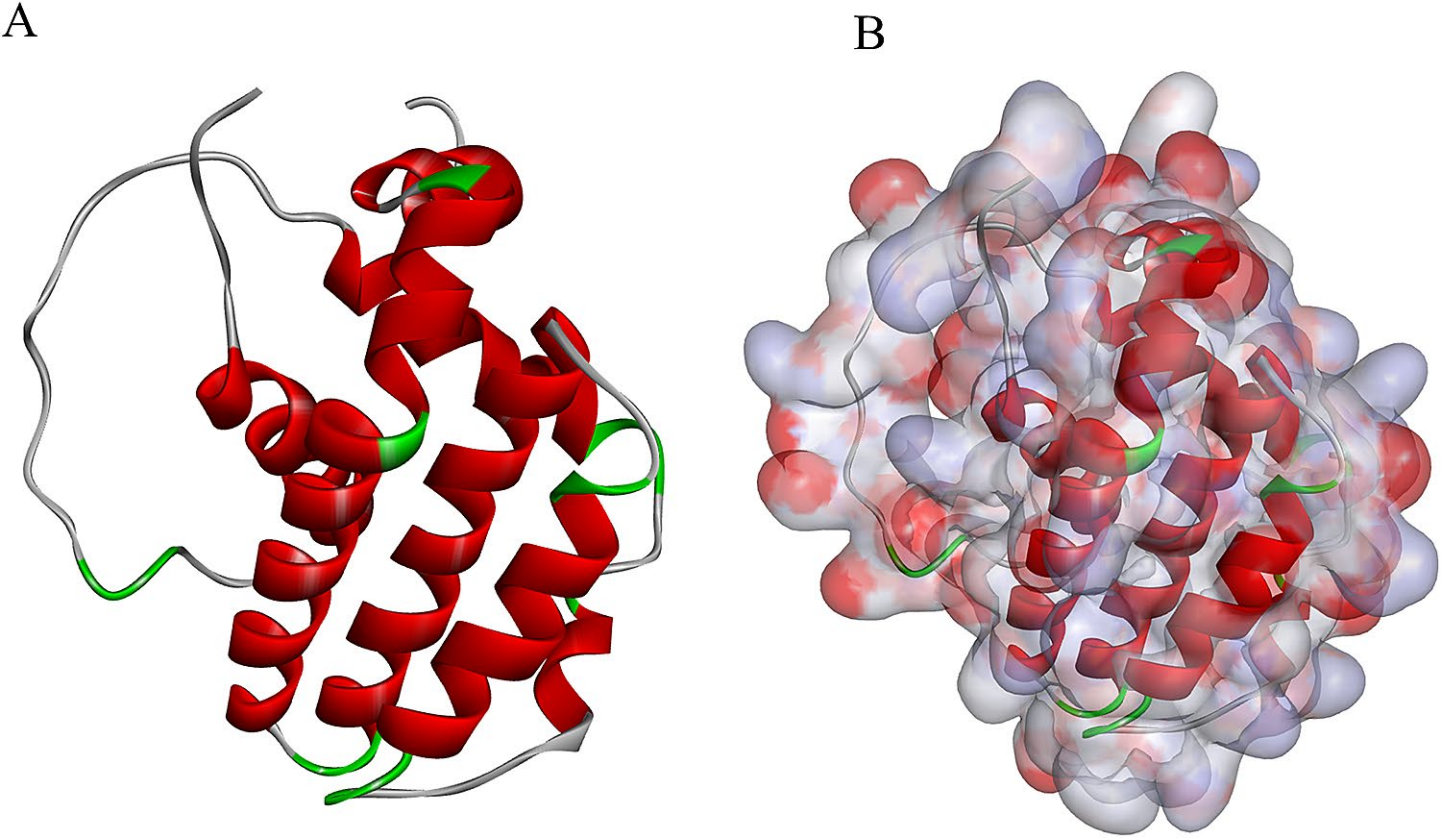
Molecular structure of BCL2. **A** Initial molecular structure. **B** Surface of binding area added. Blue represents positive charge, and red represents negative charge

Ligand-binding pocket region of BCL2 was selected as the binding site to screen compounds that could potentially inhibit BCL2. Virtual screening was carried out using the LibDock module of Discovery Studio 4.5 [21]. All ligand poses were ranked based on the ligands score. The 2.0-Å crystal structure of human BCL2 (Protein Data Bank identifier:1G5M) and the inhibitor Obatoclax (Protein Data Bank identifier: ZINC29052268) was downloaded from the Protein Data Bank and imported to the working circumstance of LibDock. The chemical structure of BCL2 is shown in Fig. 1. The protein was prepared by removing crystal water and other heteroatoms around it, followed by addition of hydrogen, protonation, ionization, and energy minimization [22]. The minimization performed 2000 steps with a root-mean-square gradient tolerance of 12.277, and the final root mean square gradient was 0.690. The prepared protein was employed to define the binding site. Using the ligands (Obatoclax) binding position, the active site for docking was generated. Based on the LibDock score, all the docked poses were ranked and grouped, and all compounds were ranked according to the LibDock score.

### Absorption, distribution, metabolism, and excretion and toxicity prediction

The ADME module of Discovery Studio 4.5 was employed to calculate absorption, distribution, metabolism, and excretion (ADME) of selected compounds, including their aqueous solubility, blood–brain barrier penetration, cytochrome P-450 2D6 (CYP2D6) inhibition, hepatotoxicity, human intestinal absorption and plasma protein binding level. TOPKAT module of Discovery Studio 4.5 was employed to calculate the toxicity and other properties of all the potential compounds. These pharmacologic properties were fully considered during the selection of proper drug candidates for BCL2.

### Molecule docking

The CDOCKER module of Discovery Studio 4.5 was used for molecular docking study. CDOCKER is a molecular docking method based on the CHARMM force field, which can produce high-precision docking results. The CHARMM force field was used for both receptors and ligands. For each complex pose, the CHARMM energy and the interaction energy, which indicated ligand binding affinity, were calculated. Crystal structure of BCL2 was obtained from the protein data bank. The crystal water molecules were generally removed in a rigid and semi-flexible docking process, causing the fixed water molecules to possibly affect the conformation of the receptor-ligand complex [23, 24]. After the water molecules were removed, hydrogen atoms were added to the protein.

### Molecular dynamics simulation

The best binding conformations of the ligand-BCL2 complexes among the poses predicted by the molecule docking program were selected and prepared for molecular dynamics simulation. To simulate the physiologic environment, sodium chloride was added to the system with the ionic strength of 0.145. Then the system was subjected to the CHARMM force field and relaxed by energy minimization (500 steps of steepest descent and 500 steps of conjugate gradient), with the final root mean square gradient of 0.227. The particle mesh Ewald algorithm was used to calculate long-range electrostatics, and the linear constraint solver algorithm was adapted to fix all bonds involving hydrogen. With initial complex setting as a reference, potential energy, and structural characteristics through the Discovery Studio 4.5 analysis trajectory protocol.

### Cell culture

The human DLBCL cell line SU‐DHL‐2 was obtained from American Type Culture Collection. The cells were cultured in high glucose DMEM medium supplemented with 10% fetal bovine serum, cultured in a 37 °C and 5% CO_2_ until the cells cover the bottom of the flask. The logarithmic growth phase of cells was selected for experimental use.

### CCK8 assay

DLBCL cell lines SU‐DHL‐2 was seeded into 96-well plates at a density of 5 × 10^3^/well, and each group had three duplicate Wells. After 24 h, Obatoclax, Beta-Hydroxyisovalerylshikonin (ZINC000002525131) and Methyl 6-hydroxyangolensate (ZINC00013298233) were added into 96-well plates at a certain concentration, and then cultured in 5%CO_2_ at 37 °C for 72 h. Add 100 μL test solution (including 10 μL CCK8 + 90 μL DMEM medium) to each well and incubate at 37 °C for 1 h. The absorbance of the solution at 450 nm was determined by an enzyme plate analyzer.

### Detection of BCL-2

Cell culture and grouping were the same as mentioned above. The operation was carried out according to the instructions of ELISA detection kit, and the BCL-2 expression of SU‐DHL‐2 cells was measured.

## Results

### Virtual screening of natural products database against BCL2

A total of 17,931 biogenic product molecules were downloaded from the ZINC database. The chemical structure of BCL2 was selected as receptor protein, and its pharmacological effects were compared with other compounds. After screening, 7326 compounds were shown to be qualified to bind firmly with BCL2. Table 1 lists the top 20 compounds. One inhibitor, Obatoclax (ZINC 29,052,268), was selected as the reference substance.

**Table 1:** Top 20 ranked compounds with LibDock scores

Number	Compounds	LibDock score
1	ZINC000005664046	144.791
2	ZINC000005922875	143.449
3	ZINC000014711612	139.392
4	ZINC000003978135	135.579
5	ZINC000003978134	134.314
6	ZINC000013451339	134.162
7	ZINC000002008850	133.248
8	ZINC000028520217	133.132
9	ZINC000003951623	132.812
10	ZINC000004175510	131.56
11	ZINC000072320087	131.328
12	ZINC000031494940	130.692
13	ZINC000001702730	130.619
14	ZINC000039066223	130.396
15	ZINC000004098005	130.337
16	ZINC000002525131	129.653
17	ZINC000013298233	129.212
18	ZINC000026489484	128.78
19	ZINC000005854691	127.26
20	ZINC000005670074	127.227

### ADME and toxicity prediction

The ADME module of Discovery Studio 4.5 was used to predict the pharmacological properties of all selected ligands and Obatoclax (Table 2). The aqueous solubility prediction (defined in water at 25 °C) showed that all the compounds were soluble in water. All compounds were predicted to be non-inhibitors with CYP2D6. For hepatotoxicity, ten compounds were found to be nontoxic, which was more harmless than Obatoclax. The residual compounds were toxic in the forecast. For human intestinal absorption, one compound, ZINC000072320087, has the highest absorption level, and eight compounds have a better absorption level than Obatoclax. Plasma protein binding properties indicated all compounds had good absorption excluding ZINC000028520217 and ZINC000072320087.

**Table 2:** Adsorption, distribution, metabolism, and excretion properties of compounds

Number	Compounds	Solubility level	BBB level	CYP2D6	Hepatotoxicity	Absorption level	PPB level
1	ZINC000005664046	2	3	0	1	0	1
2	ZINC000005922875	2	4	0	1	0	1
3	ZINC000014711612	2	4	1	0	2	1
4	ZINC000003978135	2	4	0	0	0	1
5	ZINC000003978134	2	4	0	0	0	1
6	ZINC000013451339	1	4	1	1	2	1
7	ZINC000002008850	2	4	1	0	2	1
8	ZINC000028520217	1	4	0	1	2	0
9	ZINC000003951623	2	2	1	1	0	1
10	ZINC000004175510	2	4	0	0	0	1
11	ZINC000072320087	1	4	1	1	3	0
12	ZINC000031494940	2	4	0	0	2	1
13	ZINC000001702730	2	4	0	0	0	1
14	ZINC000039066223	2	4	0	0	2	1
15	ZINC000004098005	2	4	1	1	2	1
16	ZINC000002525131	3	4	0	0	1	1
17	ZINC000013298233	2	4	0	0	0	1
18	ZINC000026489484	2	3	0	1	0	1
19	ZINC000005854691	2	4	1	1	2	1
20	ZINC000005670074	3	3	0	1	0	1
21	Obatoclax	2	1	0	1	0	1
22	Venetoclax	2	4	0	1	3	1

Aqueous-solubility level: 0, extremely low; 1, very low, but possible; 2, low; 3, good

BBB level: 0, very high penetrant; 1, high; 2, medium; 3, low; 4, undefined

CYP2D6 level: 0, noninhibitor; 1, inhibitor

Hepatotoxicity: 0, nontoxic; 1, toxic

Human-intestinal absorption level: 0, good; 1, moderate; 2, poor; 3, very poor

PPB: 0, absorbent weak; 1, absorbent strong

*BBB* blood–brain barrier; *CYP2D6* cytochrome P-450 2D6; *PPB* plasma protein binding

**Fig. 2:**
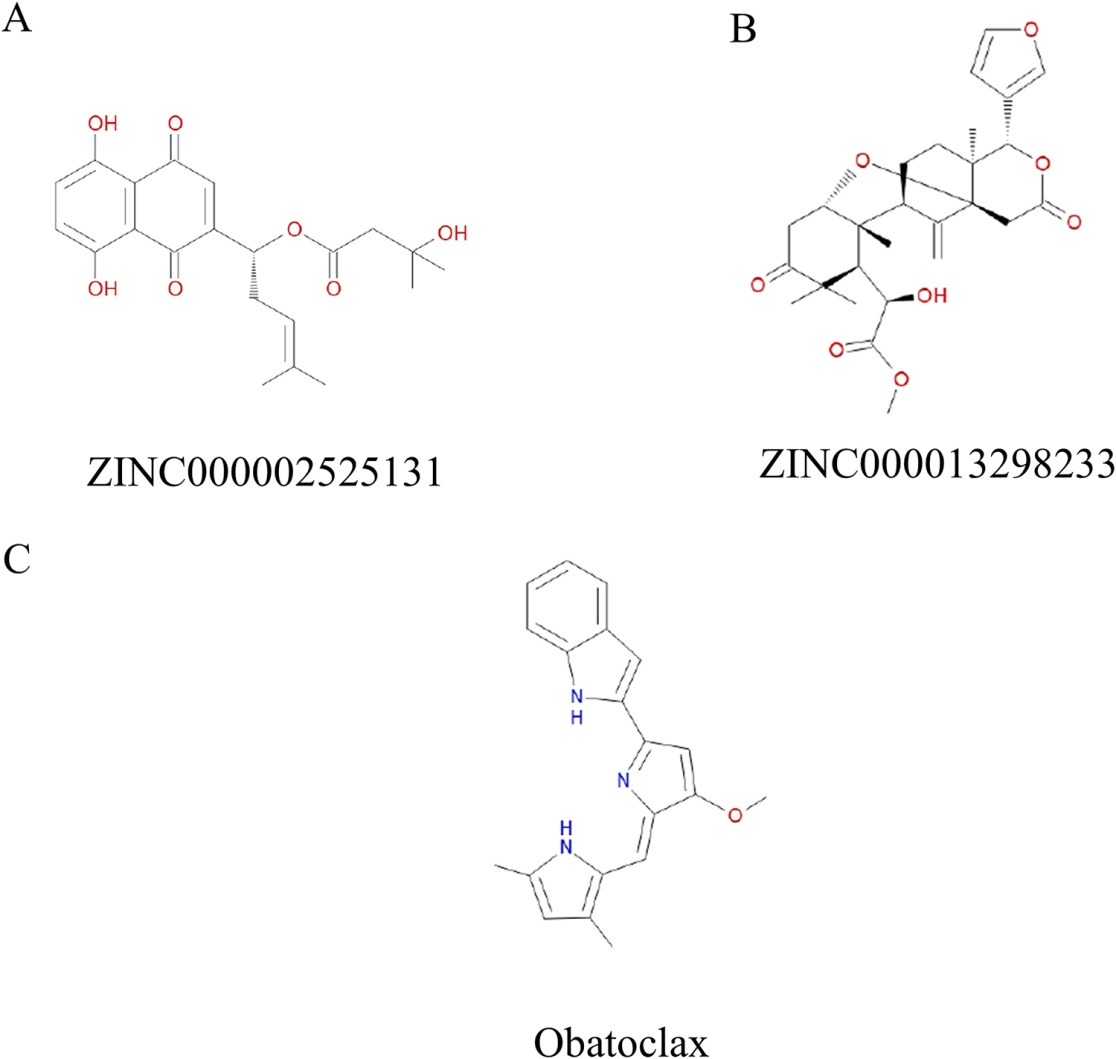
Structures of Obatoclax and novel compounds selected from virtual screening

Safety should also be entirely considered in this research. To affirm the security of the selected compounds, diverse sorts of toxicity indicators of the compounds and Obatoclax utilizing a computational way in the TOPKAT module of Discovery Studio 4.5 (Table 3). Outcomes indicated that 15 compounds were discovered to be non-mutagenic. What's more, two compounds were discovered to have no developmental toxicity potential. The reference Obatoclax was predicted to have high rodent carcinogenicity whether in mouse or rat, considering all the above results, ZINC000002525131 and ZINC000013298233 were funded to be the ideal lead compounds with non-CYP2D6 inhibitors, without hepatotoxicity. In addition, compared with other compounds, the compounds we found have less rodent carcinogenicity, Ames mutagenicity and developmental toxicity potential. In summary, ZINC000002525131 and ZINC000013298233 were proved to be safe for further study (Fig. 2).

**Table 3:** Toxicities of compounds

Number	Compounds	Mouse NTP		Rat NTP		Ames	DTP
		Female	Male	Female	Male		
1	ZINC000005664046	0	0.666	1	0.994	0	0.004
2	ZINC000005922875	0	0.965	1	0.991	0	1
3	ZINC000014711612	0.926	1	1	0.927	1	0.989
4	ZINC000003978135	0.024	1	0	1	0	0
5	ZINC000003978134	0	1	1	1	0	0.001
6	ZINC000013451339	0	0.943	0	0.038	0	1
7	ZINC000002008850	0.246	1	0.999	0.038	1	0.974
8	ZINC000028520217	0	1	0	0.415	0	0.998
9	ZINC000003951623	0	0.999	1	0.023	0	0.824
10	ZINC000004175510	0	1	1	1	0	0.001
11	ZINC000072320087	0	1	1	0.995	0	1
12	ZINC000031494940	1	0	0	0.053	0	1
13	ZINC000001702730	0	1	1	1	0	0.99
14	ZINC000039066223	0.001	1	1	0.288	1	1
15	ZINC000004098005	0	1	1	0.001	1	1
16	ZINC000002525131	0	1	1	1	0	1
17	ZINC000013298233	0.024	1	0	1	0	0
18	ZINC000026489484	0.011	0.006	0.102	0.989	0	1
19	ZINC000005854691	0	1	1	0.001	1	1
20	ZINC000005670074	0.035	0	0.996	0.564	0	1
21	Obatoclax	1	0.028	1	0.858	1	0.982
22	Venetoclax	1	1	1	0.998	0	0

NTP < 0.3(noncarcinogen); > 0.8(carcinogen)

Ames < 0.3(nonmutagen); > 0.8(mutagen)

DTP < 0.3(nontoxic); > 0.8(toxic)

*NTP* U.S. National Toxicology Program; *DTP* developmentaltoxicitypotential

### Analysis of ligand binding

To study ligand binding mechanisms of these compounds with Obatoclax, ZINC000002525131 and ZINC000013298233 were docked into BCL2 by CDOCKER module, and CDOCKER potential energy were counted and displayed as shown in Table 4. The CDOCKER potential energy of ZINC000002525131 and ZINC000013298233 was similar with the reference ligand Obatoclax (− 27.9792 kcal/mol), which illustrated that this compound had a good binding affinity with BCL2.

**Table 4:** CDOCKER potential energy of compounds with BCL2

Compounds	-CDOCKER potential energy (kcal/mol)
ZINC000002525131	43.63
ZINC000013298233	15.3924
Obatoclax	27.9792

**Fig. 3:**
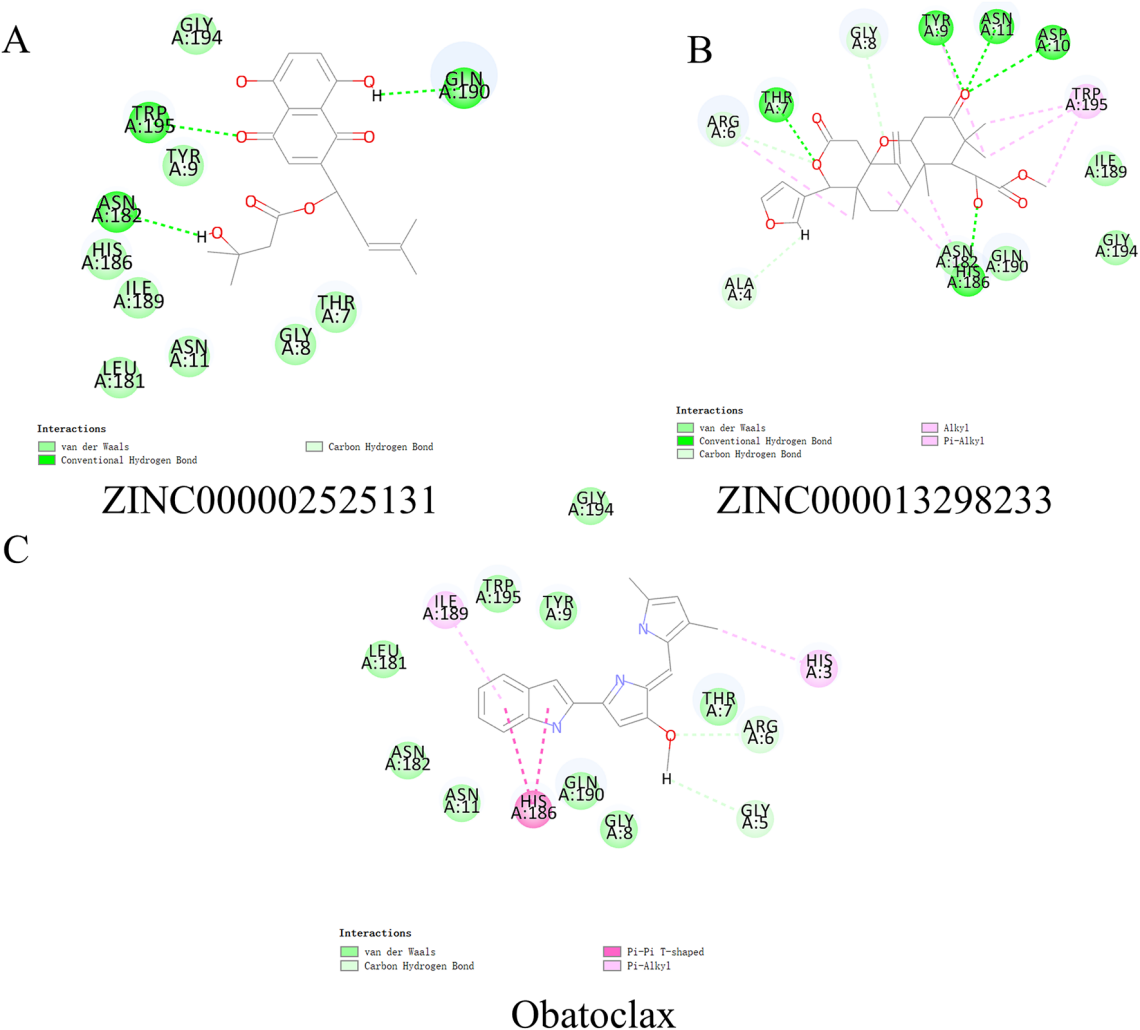
Schematic of intermolecular interaction of the predicted binding modes of **A** ZINC000002525131 with BCL2, **B** ZINC000013298233 with BCL2, and **C** Obatoclax with BCL2

**Fig. 4:**
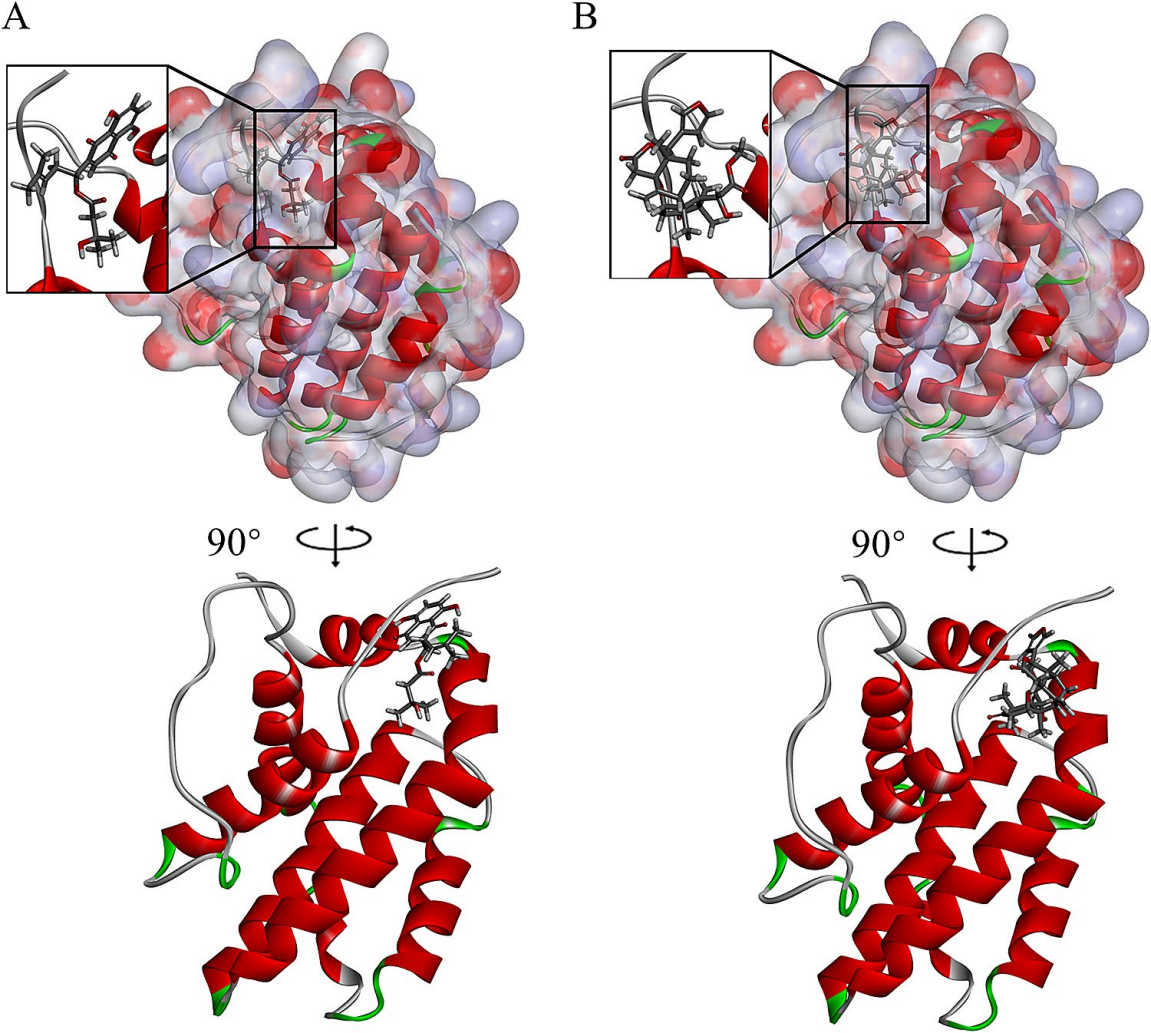
Schematic drawing of interactions between ligands and BCL2. The surface of binding areas was added. Blue represents positive charge; red represents negative charge; and ligands are shown in sticks, with the structure around the ligand-receptor junction shown in thinner sticks. **A** ZINC000002525131- BCL2 complex. **B** ZINC000013298233- BCL2 complex

Hydrogen bonds and π-related interactions were also obtained through structural computation study (Figs. 3 and 4). Results performed that ZINC000002525131 formed 15 pairs of hydrogen bonds with BCL2 (Table 5), by the O^10^ of the compound with ILE14:HN, O^181^ of the compound with HIS186:HN, O^185^ of the compound with ILE189:HN, O^189^ of the compound with GLY194:HA1 etc. Also, π-related interactions were formed in the complex (Table 6). ZINC000013298233 formed seven pairs of π-related interactions with BCL2, by TYR9 of the compound of BCL2, two pairs of HIS186 of the compound of BCL2, three pairs of TRP195 of BCL2. It also formed 18 hydrogen bonds in the complex. As for the reference compound Obatoclax, it formed five hydrogen bonds with BCL2, by the O^3^ of the compound with GLY5:HN of BCL2, the O^10^ of the compound with ILE14:HN of BCL2, O^181^ of the compound with HIS186:HN of BCL2. O^185^ of the compound with ILE189:HN of BCL2 etc. As well as 8 π-related interactions were also formed with BCL2.

**Table 5:** Hydrogen bond interaction parameters for each compound with BCL2

Receptor	Compound	Donor atom	Receptor atom	Distances (Å)
1g 5m	ZINC000002525131	ILE14:HN	ASP10:O	1.74
		HIS186:HN	LEU181:O	2.36
		HIS186:HD1	ASN182:OD1	2.1
		ILE189:HN	LEU185:O	1.68
		ILE189:HN	HIS186:O	2.56
		GLN190:HN	HIS186:O	2.26
		GLN190:HE21	HIS186:NE2	2.45
		GLY194:HN	ILE189:O	1.73
		TRP195:HN	ZINC000002525131:O18	2.41
		ZINC000002525131:H47	ASN182:OD1	2
		ZINC000002525131:H49	ZINC000002525131:O18	1.84
		ZINC000002525131:H52	GLN190:OE1	2.3
		ZINC000002525131:H52	ZINC000002525131:O28	1.93
		GLY194:HA1	ILE189:O	2.65
		TRP195:HD1	ZINC000002525131:O18	3.03
	ZINC000013298233	THR7:HN	ZINC000013298233:O14	1.59
		TYR9:HN	ZINC000013298233:O23	2.1
		ASP10:HN	ZINC000013298233:O23	2.84
		ASN11:HN	ZINC000013298233:O23	2.28
		ILE14:HN	ASP10:O	1.74
		HIS186:HN	LEU181:O	2.36
		HIS186:HD1	ASN182:OD1	2.1
		HIS186:HD1	ZINC000013298233:O29	2.31
		ILE189:HN	LEU185:O	1.68
		ILE189:HN	HIS186:O	2.59
		GLN190:HN	HIS186:O	2.26
		GLN190:HE21	HIS186:NE2	2.45
		GLY194:HN	ILE189:O	1.73
		ARG6:HA	ZINC000013298233:O14	2.7
		THR7:HB	ARG6:O	2.25
		GLY8:HA1	ZINC000013298233:O19	2.94
		GLY194:HA1	ILE189:O	2.65
		ZINC000013298233:H47	ALA4:O	2.83
	Obatoclax	GLY5:HN	HIS3:O	1.95
		ILE14:HN	ASP10:O	1.74
		HIS186:HN	LEU181:O	2.36
		HIS186:HD1	ASN182:OD1	2.1
		ILE189:HN	LEU185:O	1.68
		ILE189:HN	HIS186:O	2.59
		GLN190:HN	HIS186:O	2.26
		GLN190:HE21	HIS186:NE2	2.45
		GLY194:HN	ILE189:O	1.73
		Obatoclax:H36	Obatoclax:N15	2.3
		ARG6:HA	Obatoclax:O2	2.57
		ARG6:HD1	GLY5:O	2.74
		ARG6:HD2	GLY5:O	3.02
		THR7:HB	ARG6:O	2.25
		GLY194:HA1	ILE189:O	2.65
		Obatoclax:H26	GLY5:O	3.04

**Table 6:** $\uppi$-Related interaction parameters for each compound with BCL2

Receptor	Compound	Donor atom	Receptor atom	Distances (Å)
1g 5m	ZINC000002525131	TRP195	TYR9	4.36
		LEU181	LEU185	5.06
		TRP195	ILE14	5.25
		TRP195	ILE14	4.35
	ZINC000013298233	ZINC000013298233:C7	ARG6	4.08
		TYR9	ZINC000013298233:C26	5.01
		HIS186	ZINC000013298233	5.45
		HIS186	ZINC000013298233:C35	4.43
		TRP195	ZINC000013298233:C25	5
		TRP195	ZINC000013298233:C26	4.33
		TRP195	ZINC000013298233:C33	5.24
	Obatoclax	TRP195	TYR9	4.36
		HIS186	Obatoclax	4.91
		HIS186	Obatoclax	4.94
		LEU181	LEU185	5.06
		HIS3	Obatoclax:C24	4.29
		TRP195	ILE14	5.25
		TRP195	ILE14	4.35
		Obatoclax	ILE189	5.06

### Molecular dynamics simulation

**Fig. 5:**
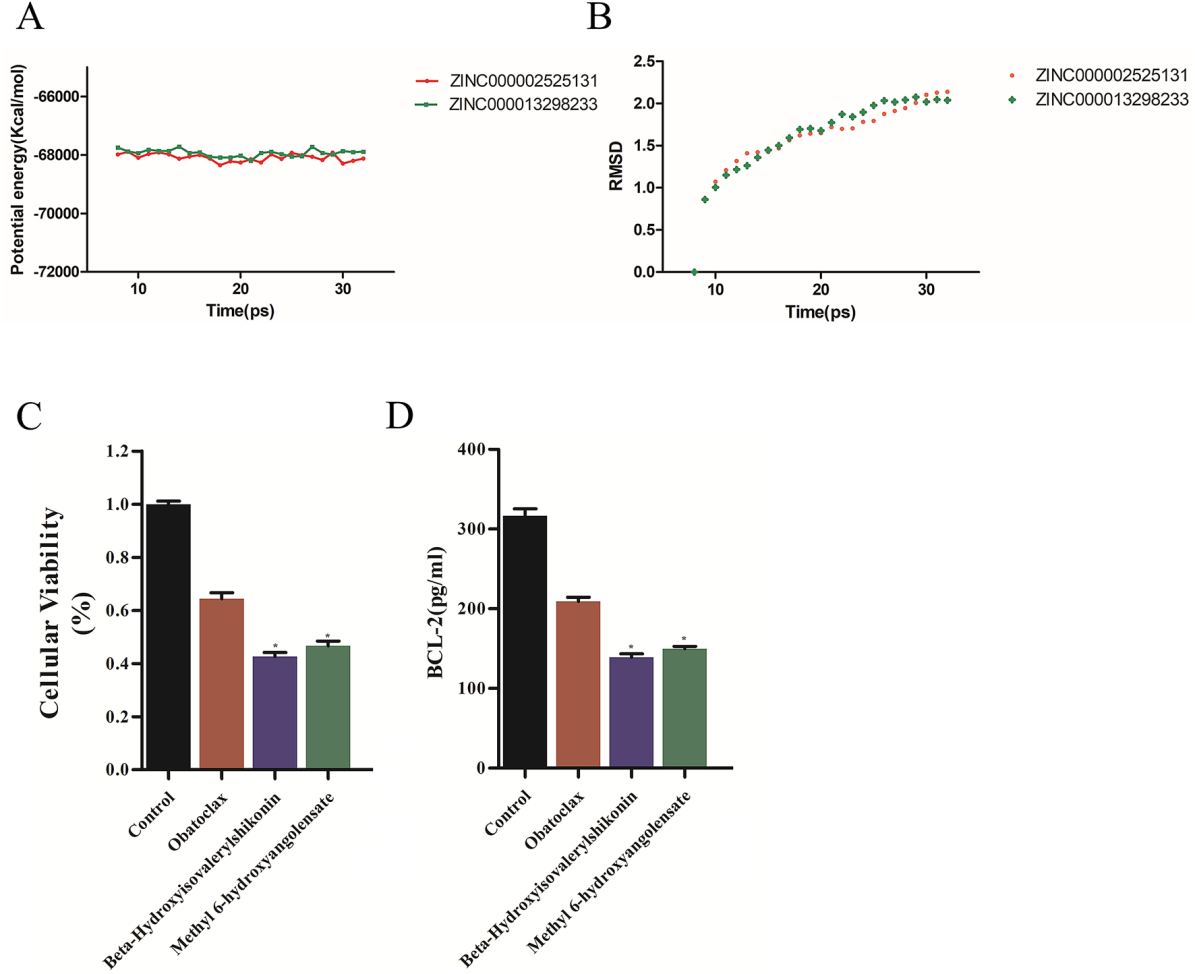
Results of molecular dynamics simulation of the compounds ZINC000002525131 and ZINC000013298233. **A** Potential energy, average backbone root-mean-square deviation. **B** RMSD, root-mean-square deviation. **C** Cellular viability of SU‐DHL‐2 cells. **D** BCL-2 expression in SU‐DHL‐2 cells

The stability of ligand-BCL2 complexes was simulated under the natural environment by molecular dynamics simulation. The RMSD curves and potential energies of the complexes were calculated by molecular docking experiments using CDOCKER module (Fig. 5A, B). After 18 ps, the trajectories of complex reached balance; RMSD and potential energy of these complexes stabilized with time. Results showed that these two compounds could interact with BCL2, and in the natural environment, their complexes could exist stably.

### Experiment to verify the therapeutic effect of the two selected compounds on the viability of SU‐DHL‐2 cells and BCL-2 expression in SU‐DHL‐2 cells

The SU‐DHL‐2 cells were treated with Obatoclax, Hydroxyisovalerylshikonin and Methyl 6-hydroxyangolensate for 72 h. Then the cell viability was detected by CCK8 kit. The results showed that Obatoclax, Hydroxyisovalerylshikonin and Methyl 6-hydroxyangolensate had an inhibitory effect on the proliferation of SU‐DHL‐2 cells compared with the blank control group. The cell viability of Obatoclax, Hydroxyisovalerylshikonin and Methyl 6-hydroxyangolensate group was smaller than that of blank group, and the inhibitory effect of Hydroxyisovalerylshikonin and Methyl 6-hydroxyangolensate group on the proliferation of SU‐DHL‐2 cells was stronger than that of Obatoclax group (Fig. 5C).

Additionally, the level of BCL-2 secretion in, Obatoclax, Hydroxyisovalerylshikonin and Methyl 6-hydroxyangolensate group was found lower than that in blank group, and the level of BCL-2 secretion in Hydroxyisovalerylshikonin and Methyl 6-hydroxyangolensate group was lower than that in Obatoclax group. (Fig. 5D).

## Discussion

DLCBL is one of the most common malignant lymphomas in adults, which account for 24% new cases in non-Hodgkin lymphoma [1]. This disease is highly aggressive and needs treatment rapidly. Research shows that DLCBL is highly related to BCL2. BCL-2 protein mainly controls cell apoptosis by regulating the direct binding interaction of mitochondrial outer membrane permeabilization (MOMP), resulting in irreversible release of space proteins between membranes, followed by caspase activation and apoptosis. The key point to suppress tumor growth is to find an inhibitor of BCL2 to restrict its activity so as to resist tumor growth. Therefore, there is an urgent need to expose effective BCL2 targeted drugs.

Recently, the development of new BCL2 inhibitors for clinical application, combined with various anticancer drugs to enhance the therapeutic effects, has become a research highlight in the field of anti-tumor drugs. Although great progress has been made in the design and development of BCL2 inhibitors drug, there are still many limitations in regard to the inhibitors. It is urgent to find more BCL2 compounds for chemotherapy and clinical applications. In this study, Obatoclax was chosen as a reference drug.

In this study, 17,931 biological products were extracted from the ZINC15 database for virtual screening, followed by ADME, TOPKAT, CDOCKER, and molecular dynamics simulation. The degree of energy optimization and conformational stability can be shown by LibDock scores. The compounds with higher LibDock scores showed energy optimization and conformational stability compared with other compounds. Through the calculation of the LibDock module, we found that 6326 compounds could bind to BCL2 stably. The top 20 natural compounds were chosen and collected for further research.

ADME and toxicity prediction were carried out to evaluate the pharmacological properties of the selected compounds. Results showed that ZINC000002525131 and ZINC000013298233 were identified as ideal lead compounds because they had an excellent intestinal absorption level and were soluble in water. Moreover, they were predicted to be non-inhibitors of CYP2D6 and non-hepatotoxic. Compared with other compounds, the carcinogenicity, Ames mutagenicity and developmental toxicity of these two compounds were lower in rodents, which illustrated that 2 compounds had good safety for as potential ideal lead compounds. These data illustrate their application prospects in the field of drug designation, as it is safer and more effective than Obatoclax. Although the remaining drugs on the list possess toxicities or negative effects, they can reduce the toxicity by adding or deleting specific groups and atoms, and they also have potential application prospects in drug development. Considering all the above reported results, ZINC000002525131 and ZINC000013298233 were selected as ideal lead compounds, and further analysis was carried out.

Venetoclax, also known as ABT-199, is a highly potent, orally bioavailable and BCL-2–selective inhibitor [25]. Previous research has been done to bind Venetoclax with BCL2 with a predicted binding free energy of − 10.24. Docking calculation exposed that Venetoclax binds with common interacting residues F104, R107, Y108, and G145. Q99 residue of physiological BCL-2 form makes two hydrogen bonds with N15 with the distance of 3.14 Å and O40 atoms with the bond distance of 2.9 Å of Venetoclax [26]. In our research, ZINC000002525131 and ZINC000013298233 have lower binding free energy as − 43.63 kcal/mol and − 15.3924 kcal/mol and more hydrogen bonds, which shows better stability than Venetoclax. Besides, we performed ADME and toxicity prediction of Venetoclax. Venetoclax showed hepatotoxicity and high toxicities in female rats, female mice, male rats and male mice, respectively, which are also poor than the two compounds we selected.

The ligand binding mechanism and chemical bond between the candidate compounds and BCL2 were found. CDOCKER module calculation demonstrated that the CDOCKER interaction energies of zinc00002525131 and zinc00013298233 were significantly lower than Obatoclax, which indicates that the binding affinity of these two compounds to BCL2 may be higher than that of Obatoclax. Next, the structures of the complexes of compounds and BCL2 were examined. The molecular dynamics simulation analysis showed that the complexes also can exist in a natural environment steadily, which is suitable for a potential medicine.

Then, CCK8 assay and ELISA in vitro were carried out to evaluate the effects of potential compounds in the study. We chose the secretion level of BCL-2 and the proliferation level of DLCBL cell as the evaluation indicators to access the drug effect. In ELISA assay, the level of BCL-2 secretion in Beta-Hydroxyisovalerylshikonin and Methyl 6-hydroxyangolensate group was lower than that in Obatoclax group. In CCK8 assay, the cellular viability in cell lines SU‐DHL‐2 treated with Beta-Hydroxyisovalerylshikonin and Methyl 6-hydroxyangolensate were smaller than that of Obatoclax. Therefore, the results demonstrated that the effect of Hydroxyisovalerylshikonin and Methyl 6-hydroxyangolensate was better that of Obatoclax in anti- DLCBL.

At the present time, design and development of cancer drugs were topics attracting worldwide attention, although overall progress seems not encouraging. This research demonstrates that the most significant step in current drug design was to screen ideal lead compounds. The results showed that the selected compounds were comprehensively examined to determine their superiority over the reference compound Obatoclax. After a series of computational studies, these two compounds may be the most potential drugs for the treatment of diffuse large B-cell lymphoma. However, it is worth noting that these drugs must be improved in the experiment before they can be used clinically. In the future, these two compounds can be further improved and refined so that they can be applied. Moreover, our research provides guidance for choosing lead compounds that may have potential influence. The high-tech method promotes the development of current drugs.

Although the research was conducted through careful design and accurate measurement, there are still some limitations. Further experiments, such as animal experiments, are needed to verify our results more reliably. In the future research, it is necessary to research the half-maximal inhibitory concentration and half-maximal effective concentration.

## Conclusions

This research performed a series of computer-aided structural and chemistry techniques (e.g., virtual screening, molecule docking ADME, toxicity prediction) to find and identify the ideal lead compounds with functions to potentially inhibit BCL2. Two compounds, ZINC000002525131 and ZINC000013298233, were chosen as safety drug candidates, and they had great importance in BCL2 inhibitor development. In addition, this study supplies a series of candidate drugs with pharmacological properties, which provides a solid foundation for drug design and improvement of BCL2.
